# Clinical manifestation and genetic findings in three boys with low molecular Weight Proteinuria - three case reports for exploring Dent Disease and Fanconi syndrome

**DOI:** 10.1186/s12882-020-02225-6

**Published:** 2021-01-11

**Authors:** Nan Duan, Chenwei Huang, Lu Pang, Shiju Jiang, Wenshuang Yang, Haixia Li

**Affiliations:** Department of Clinical Laboratory, Peking University First Hospital, No.8 Xishiku St., Xicheng District, 100034 Beijing, China

**Keywords:** Dent disease, Fanconi syndrome, Low molecular weight proteinuria, Genetic analysis, Case report

## Abstract

**Background:**

Dent disease is an X-linked form of progressive renal disease. This rare disorder was characterized by hypercalciuria, low molecular weight (LMW) proteinuria and proximal tubular dysfunction, caused by pathogenic variants in *CLCN5* (Dent disease 1) or *OCRL* (Dent disease 2) genes. Fanconi syndrome is a consequence of decreased water and solute resorption in the proximal tubule of the kidney. Fanconi syndrome caused by proximal tubular dysfunction such as Dent disease might occur in early stage of the disease.

**Case presentation:**

Three cases reported in this study were 3-, 10- and 14-year-old boys, and proteinuria was the first impression in all the cases. All the boys presented with LMW proteinuria and elevated urine albumin-to-creatinine ratio (ACR). Case 1 revealed a pathogenic variant in exon 11 of *CLCN5* gene [NM_001127899; c.1444delG] and a nonsense mutation at nucleotide 1509 [p.L503*], and he was diagnosed as Dent disease 1. Case 2 carried a deletion of exon 3 and 4 of *OCRL1* gene [NM_000276.4; c.120-238delG^…^A] and a nonsense mutation at nucleotide 171 in exon 5 [p.E57*], and this boy was diagnosed as Dent disease 2. Genetic analysis of Case 3 showed a missense mutation located in exon 2 of *HNF4A* gene [EF591040.1; c.253C > T; p.R85W] which is responsible for Fanconi syndrome. All of three pathogenic variants were not registered in GenBank.

**Conclusions:**

Urine protein electrophoresis should be performed for patients with proteinuria. When patients have LMW proteinuria and/or hypercalciuria, definite diagnosis and identification of Dent disease and Fanconi syndrome requires further genetic analyses.

## Background

Dent disease (OMIM #300009) is a rare proximal renal tubular dysfunction that occurs mostly in males. It is a group of X-linked inherited disorders characterized by low molecular weight (LMW) proteinuria, hypercalciuria, nephrocalcinosis, nephrolithiasis and progressive renal dysfunction leading to chronic kidney disease (CKD). Additional tubular defects, such as hyperphosphaturia, glycosuria, aminoaciduria, uricosuria and kaliuresis may also be observed in Dent disease which is often complicated by osteomalacia or rickets in a minority of patients [1]. In addition, this disease can also present as a generalized renal proximal tubulopathy (i.e., renal Fanconi syndrome). Two genetic subtypes of Dent disease have been described to date: Dent disease 1 (OMIM #300008) is caused by pathogenic variants of Chloride voltage-gated channel 5 (*CLCN5*) gene which maps on chromosome Xp11.22, encoding a 746 amino-acidelectrogenic Cl^−^/H^+^ exchanger (ClC-5), and Dent disease 2 (OMIM #300555) is caused by pathogenic variants of Oculocerebrorenal syndrome of Lowe *(OCRL)* gene, located on chromosome Xq25, encoding inositol polyphosphate 5-phosphatase [2–4]. In addition to Dent disease, *CLCN5* associated disease have a variable phenotype including focal segmental glomerulosclerosis (FSGS), and the *OCRL* path variants are also associated with Oculocerebrorenal syndrome. About 50%-60% of patients harbor inactivating pathogenic variants of the *CLCN5* gene (Dent disease 1), about 15% have the *OCRL* pathogenic variants (Dent disease 2), and other yet unidentified genes are likely involved in Dent disease [1, 5]. Patients usually have manifestations of Dent disease from early childhood or young adult years [6]. Occasionally, patients present with either CKD or end-stage kidney disease (ESKD) are diagnosed retrospectively. LMW proteinuria is present in almost all affected males and some symptomatic female carriers. Affected males often have aminoaciduria, phosphaturia, and glycosuria. Most male patients develop CKD, with the substantial decline in glomerular filtration rate (GFR). Heterozygous females usually have only proteinuria and mild hypercalciuria [7].

The pathogenic factors of primary proximal tubulopathies include X-linked hypercalciuric nephrolithiasis (caused by a pathogenic variant in Chloride channel gene *CLCN5*), mutation in the ubiquinol-cytochrome c reductase synthesis-like (*BCS1L*) gene, classic Fanconi syndrome, etc. Fanconi syndrome (OMIM #134600) presents as a generalized dysfunction of renal proximal tubule, characterized by a reabsorption defect in kidney, resulting in immoderate urinary wasting of amino acids, phosphate, glucose, bicarbonate, and other solutes. The common clinical manifestations are polyuria, polydipsia, failure to thrive, as well as renal salt wasting, metabolic acidosis, calciuria and phosphaturia [8]. Most of these abnormalities will affect bone deposit and thus growth, leading to the development of rickets, osteoporosis or osteomalacia [9]. Fanconi syndrome may be inherited or acquired [10]: it often occurs as part of a multisystem metabolic disease from genetic deficiencies in children and typically presents as an acquired type in adults [7, 11]. The prognosis for this disease is not optimistic, with half of the patients progressing to CKD [11]. The glomerular filtration rate (GFR) invariably decreases and ESKD occurs in late childhood if untreated [7, 11].

Here, we report three cases with proteinuria as a common clinical manifestation but different genetic pathogenic variants: proteinuria was showed as the first impression in all three boys presented with LMW proteinuria and albuminuria. Case 1 was diagnosed as Dent disease 1 with a *CLCN5* pathogenic variant, Case 2 was diagnosed as Dent disease 2 carrying the pathogenic variant in the *OCRL* gene (initially diagnosed with Fanconi syndrome according to clinical manifestations), and Case 3 was consistent with Fanconi syndrome caused by hepatocyte nuclear factor 4α (*HNF4A*) pathogenic variant.

## Case presentation

The clinical and laboratory data of three boys were analyzed retrospectively. Genomic DNA was extracted from their peripheral blood, analyzed by Sanger DNA sequencing in the genetic laboratory of Running Gene Inc. in Beijing, China. The genetic data of Case 2 was confirmed by quantitative real-time polymerase chain reaction (PCR). We performed Nucleotide BLAST program to find regions of local similarity between the sequences of three variants and the sequences in the GenBank nucleotide databases at U.S. National Library of Medicine. Criteria and guidelines proposed by the American College of Medical Genetics and Genomics (ACMG) were used for the interpretation of sequence variants and the classification/determination of the variant pathogenicity [12].

### Case 1

A 3-year-old boy was admitted to Peking University First Hospital (June 14, 2018) with complaints of polydipsia, polyuria, and proteinuria for 5 months. Laboratory examination suggested that he had LMW proteinuria without hematuria, hypertension, or renal dysfunction. Renal biopsy was performed in a local hospital in March, 2018. Glomerular minor lesion was observed under light microscope, and minimal glomerular lesion was diagnosed by electron microscopy.

**Fig. 1:**
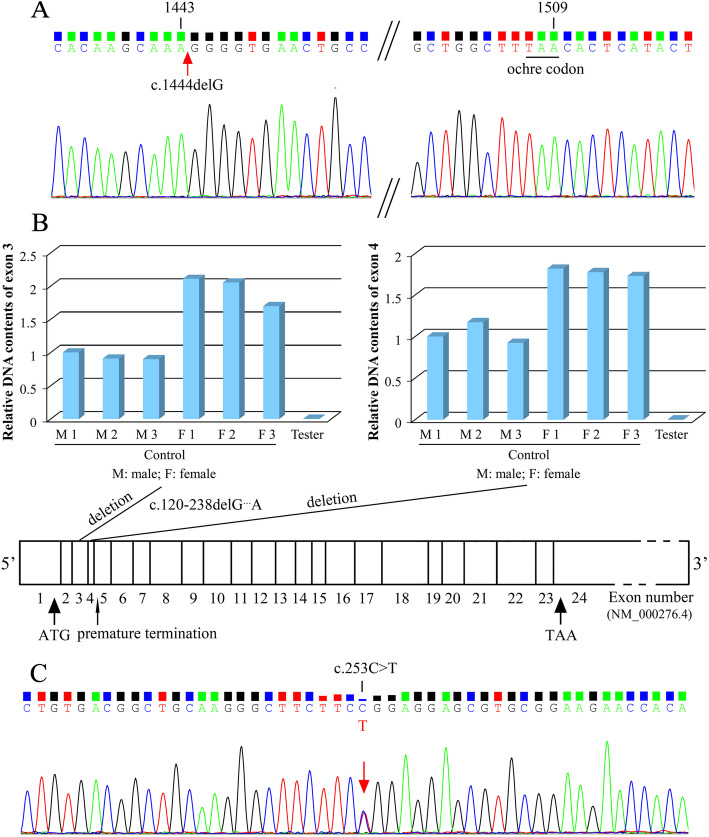
Results of genetic analyses in three cases . **a.** Deletion mutation (arrow) in exon 11 of the *CLCN5* gene [c.1444delG] and a nonsense mutation at nucleotide 1509 [p.L503*] in Case 1. **b.** Relative DNA contents of exons 3–4 of human *OCRL1* gene and schematic representation of exonic location of the identified deletion mutations (c.120-238delG…A) and nonsense mutation at nucleotide 171 in exon 5 in the mutant OCRL1 gene [p.E57*] in Case 2. **c.** Missense mutation in exon 2 of *HNF4A* gene (arrow) [c.253C > T; p.R85W] in Case 3

At this admission, his height was 101 cm, weight was 17.7 kg, and blood pressure was 87/46 mmHg. Laboratory investigations revealed that he had proteinuria (2+) with a 24-hour urinary total protein (UTP) beyond normal range, albuminuria (increased urine albumin and albumin-to-creatinine ratio [ACR]), tubular proteinuria (LMW proteinuria and elevated urine α1-microglobin [α1-MG]) and hypophosphataemia but no hypercalciuria(Table 1). Renal ultrasonography showed no obvious abnormality. DNA sequencing showed a deletion of G at nuleotide 1444 in exon 11 of the *CLCN5* gene [NM_001127899; c.1444delG] and the deletion led to a downstream ochre codon (TAA) mutation at nuleotide 1509 in exon 11 [p.L503*] (pathogenic, Dent disease 1, X-linked recessive) in this child **(**Fig. 1A**)**. The pathogenic criterion is weighted as PVS1 (very strong evidence of pathogenicity) null variant [12]. This nonsense mutation was inherited from his mother and he was diagnosed as Dent disease 1.

**Table 1:** General information and results of the biochemical investigations performed in three children

Items	Case 1	Case2	Case3	
Gender	Male	Male	Male	
Current age (years)	3	10	14	
Height (cm)	101	127	152	
Weight (kg)	17.7	26.4	34.7	
Blood pressure (mmHg)	87/46	94/54	114/88	
Chief complaint	proteinuria, polydipsia and polyuria	proteinuria	proteinuria, glycosuria and hypercalciuria	
**Serum investigations**				reference range
Albumin (g/L)	46.5	44.1	43.2	40.0–55.0
Prealbumin (mg/L)	163.3	154.2	153.3	200.0-400.0
Sodium (mmol/L)	135.89	133.57	140.70	137.00-147.00
Potassium (mmol/L)	3.65	2.95	3.64	3.50–5.30
Chlorine (mmol/L)	102.3	100.2	108.0	99.0-110.0
Calcium (mmol/L)	2.56	2.45	2.31	2.11–2.52
Phosphate (mmol/L)	1.40	1.41	1.29	1.45–2.10
Cholesterol (mmol/L)	4.43	3.30	4.06	3.40–5.20
Creatinine (µmol/L)	30.5	48.9	105.1	36.2–52.9 (3y) 44.7–67.7 (10-11y) 52.2–92.8 (14-16y)
24-h Ccr (ml/min)	58.09	59.95	44.26	80–120
eGFR (mL/min/1.73 m^2^)	142.61	107.48	63.25	
Uric acid (µmol/L)	236	267	94	150–420
Urea (mmol/L)	4.04	7.34	5.05	1.80–7.10
**Urine investigations**				reference range
Protein	2+	2+	2+	negative
UTP (g/24 h)	0.50	1.28	0.92	0-0.15
Albumin (mg/L)	165	277.00	95.20	0–19.00
ACR (mg/g)	291.72	422.19	131.49	0–30.00
α1-MG (mg/L)	334.00	416.00	260.00	0–12.00
Protein Electrophoresis (%)				negative
LMW protein	46.5	46.9	9.5	
Albumin	38.5	34.0	35.6	
HMW protein	15.0	19.1	54.9	
UCa/Ucr (g/gcr)	0.19	0.34	0.50	0-0.20
Calcium (mmol/24 h)	0.63	7.86	8.32	2.5–7.50
Phosphate (mmol/24 h)	10.17	13.55	29.57	9.7–42

### Case 2

A 10-years boy was referred to our hospital (Dec 11, 2018) because of proteinuria for 4 years. The patient was found to have proteinuria 4 years ago due to scarlet fever in a local hospital (Sept 14, 2014). This child had LMW proteinuria, hypercalciuria, hyperphosphaturia, aminoaciduria as previous urinalysis revealed.

At admission, his height was 127 cm, weight was 26.4 kg and blood pressure was 94/54 mmHg. He had a relatively low height (percentile value for 10-years boy: 3rd, 128.7 cm) and weight (percentile value for 10-years boy: 10th, 26.55 kg), and his cardiopulmonary function, nervous system, and neurocognition are normal. Laboratory investigations showed proteinuria (2+) with increased 24-hour UTP, albuminuria (elevated urine albumin and ACR), tubular proteinuria (LMW proteinuria and elevated urine α1-MG), hypercalciuria (increased urinary calcium to creatinine ratio [UCa/Ucr]), hypophosphatemia and hypokalemia (Table 1**)**. Renal ultrasonography showed crystallization (a suspected calculus) of right kidney (0.4 cm in diameter). Sanger’s DNA sequencing showed this boy carried a hemizygous deletion of exon 3 and 4 in *OCRL1* gene [NM_000276.4; c.120-238delG^…^A], and the deletion led to a nonsense mutation and premature termination at nucleotide 171 in exon 5 of the mutant *OCRL1* gene [p.E57*] (pathogenic, Dent disease 2, X-linked recessive) (Fig. 1B**)**. The pathogenic criterion is weighted as PVS1 null variant [12], and he was diagnosed as Dent disease 2.

### Case 3

A 14-years boy was hospitalized to our hospital (Aug 14, 2018) with a complaint of proteinuria, glycosuria and hypercalciuria for 11 years. His abdominal CT scan showed nephrocalcinosis in right kidney 2 years ago. The patient was treated at a local hospital for unstable walking and ankle swelling at the age of 2 with proteinuria, glycosuria and aminoaciduria.

At this admission, his height was 152 cm, weight was 34.7 kg and blood pressure was 114/88 mmHg. Laboratory investigations revealed elevated creatinine with decreased 24-hour creatinine clearance (24-h Ccr) and estimated glomerular filtration rate (eGFR), proteinuria (2+) with elevated 24-hour UTP, albuminuria (elevated urine albumin and ACR), tubular proteinuria (LMW proteinuria and elevated urine α1-MG), hematuria (urine red blood cells of 5–8/HP), hypercalciuria (increased UCa/Ucr), glycosuria (4+) in the absence of hyperglycemia, hypouricemia, and hypophosphatemia (Table 1). X-ray examination suggested osteoporosis. Renal ultrasonography and CT scan showed nephrocalcinosis. Sanger DNA sequencing revealed a missense mutation located in exon 2 of *HNF4A* gene [EF591040.1; c.253C > T; p.R85W] (pathogenic, Fanconi syndrome, autosomal dominant) (Fig. 1C) (his parents did not carry the same variant), and he was diagnosed as Fanconi syndrome. The segregation revealed that the pathogenic criterion is weighted as PS2 (strong evidence of pathogenicity) de novo variant [12].

All of the pathogenic variants were not registered in GenBank.

## Discussion and conclusions

In this report, the three cases presented with LMW proteinuria, elevated urine ACR and α1-MG. Case 1 carried pathogenic variant in exon 11 of the *CLCN5* gene, and he was diagnosed as Dent disease 1. Case 2 carried the deletion of exon 3 and 4 in the *OCRL1* gene and he was diagnosed as Dent disease 2. Case 3 had a missense pathogenic variant in the *HNF4A* gene which is responsible for Fanconi syndrome. The three pathogenic variants were not registered in the GenBank nucleotide databases. As a result, similar clinical manifestation but different genetic pathogenic variants were found in two cases of Dent disease and one case of Fanconi syndrome. For patients with proteinuria and/or hypercalciuria, urine protein electrophoresis should be performed to clarify the composition of urine proteins. And genetic analyses would confirm the identification and diagnosis.

Dent disease (OMIM #300009) is a rare disorder affecting around 250 and 90 families which were respectively reported by Devuyst O et al. in 2010 [13] and by Mansour-Hendili L et al. in 2015 [1]. This X-linked progressive renal disease was characterized by hypercalciuria, LMW proteinuria and proximal tubular dysfunction. The clinical diagnosis of Dent disease requires the presence of all of the three following criteria: LMW proteinuria; hypercalciuria; and at least one of the following indicators: nephrocalcinosis, kidney stones, hypophosphataemia, hematuria or renal insufficiency [13]. The clinical diagnosis is supported by a history of X-linked inheritance of renal Fanconi syndrome and/or nephrolithiasis, and confirmed by the identification of pathogenic variants in either the *CLCN5* (Dent disease 1) or *OCRL* (Dent disease 2) genes that are located on chromosome Xp11.22 and Xq25, respectively. In addition, as the CLCN5 gene and OCRL gene are both located on X-chromosome, the zygosity of the gene variants is hemizygous in males. Most patients have a defect in the renal ClC-5 chloride channel due to the pathogenicity of the mutations (pathogenic variants). Lack of the ClC-5 channel activity interferes with protein resorption function of the tubule through the megalin-cubilin receptor system and cell surface receptor recycling and may explain the proteinuria, phosphaturia, glycosuria in Dent disease [3, 7]. Fanconi syndrome (OMIM #134600) is a consequence of decreased water and solute reabsorption in the proximal tubule of the kidney. Patients have polydipsia and polyuria with phosphaturia, glycosuria, and aminoaciduria. Fanconi syndrome is caused by proximal tubular dysfunction such as Dent disease and usually occurs in early stage of the disease. The link between *HNF4A* p.R63W (also described as p.R76W or p.R85W) mutation and Fanconi syndrome was initially reported in 2014, a novel atypical cause of autosomal dominant Fanconi syndrome caused by this *HNF4A* mutation on chromosome 20q [14].

Proteinuria, especially nephrotic-range proteinuria, is easily confused with other renal diseases, such as nephrotic syndrome. Therefore, high clinical attention is vital in investigating children with proteinuria since renal tubulopathy may be misinterpreted for glomerular injury, and relevant examinations are needed to determine whether it is glomerular proteinuria or tubular proteinuria. 24-hour UTP in patients with renal syndrome is usually greater than 3.5 g/24 h (mainly glomerular proteinuria), while renal tubular proteinuria is less than 1.5 g/24 h. For patients with proteinuria and normal serum albumin, renal tubular disease should be first considered [15]. Therefore, we recommend that urine protein electrophoresis could be considered for patients who need to further clarify the composition of urine proteins (such as our three cases with UTP < 1.5 g/24 h). Urinary proteins can be examined by protein electrophoresis (which could identify the distribution of LMW protein, albumin and high molecular weight protein [HMW]) and differentiated on the basis of molecular weight. The HMW proteins (> or = 69 kD) and albumin (68 kD) were considered indicative of glomerular origin, whereas LMW proteins (< 69 kD) were of tubular origin. Urine protein electrophoresis is effective for finding LMW proteinuria. LMW proteinuria in Dent disease can be confirmed by measuring urinary excretion of proteins such as α1-MG and β2-microglobulin (β2-MG), retinol-binding protein, Clara cell protein, and vitamin D binding protein [13, 16]. In this report, Case 1 (Dent disease 1) and Case 2 (Dent disease 2) both had extremely high LMW proteinuria (over than 45%), indicating renal diseases cause proximal tubular dysfunction. Some nephrologists would prefer direct testing for LMW proteins such as α1-MG or β2-MG over urine protein electrophoresis, because these test are easier to perform and more widely available. A recent study carried out in Chinese pediatric patients with renal tubular and interstitial diseases suggested that the ratio of urinary α1-MG to albumin > 1 could be used as a diagnostic criterion for tubuloproteinuria [17]. Consistent with this report, urinary α1-MG to albumin in these three cases were all > 1, which might have some benefits for early diagnosis. In contrast to Case 1, Case 2 and Case 3 also had hypercalciuria. It is reported [18] that LMW proteinuria does not produce any systemic symptoms whereas hypercalciuria can cause nephrolithiasis or nephrocalcinosis as the course progresses, which was indeed observed in Case 3 (Fanconi syndrome) at the age of 12. Thus we thought his renal function was low perhaps because he had progressive nephrocalcinosis as a result of the hypercalciuria.

Dent disease may present with clinical features of Fanconi syndrome. In the current study, both Case 2 and Case 3 were initially diagnosed with Fanconi syndrome, and genetic test further confirmed that the Case 2 was actually Dent disease 2. A previous study suggested Fanconi syndrome caused by primary renal proximal tubulopathy (such as Dent disease) usually occurs in early stage of the disease [18]. In Japan, children with Dent disease were often found during annual urinary screening which could improve early detection and intervention, and few of them had features of Fanconi syndrome [19]. We assumed that early intervention might reduce the incidence of severe tubular dysfunction in children with Dent disease in Japan. Thus, urine protein electrophoresis should be performed for patients with proteinuria to achieve early diagnosis and treatment. Moreover, Dent disease should be taken into consideration when patients with Fanconi syndrome have LMW proteinuria and hypercalciuria. It is well known that constant proteinuria itself is a damaging factor for the renal system. Unlike other two cases, Case 3 had not only proteinuria but also hematuria and elevated serum creatinine with decreased 24-h Ccr and eGFR. In addition, the urinary electrophoresis showed that he had 9.5% of LMW protein but 54.9% of HMW protein which could reflect glomerular injury. The findings might indicated that the renal tubulopathy worsened, glomerular injury emerged, and serum creatinine increased, leading to deterioration of renal function as the course progresses in Fanconi syndrome.

The nonsense mutations of *CLCN5* and *OCRL1* genes and the misense mutation of *HNF4A* gene were observed in the present report. All of three pathogenic variants of mutations were not registered in the GenBank up to present. Case 1 carried the pathogenic variants in the *CLCN5* gene located on chromosome Xp11.22, which are the cause of Dent disease 1, and case 1 was diagnosed as Dent disease 1. *CLCN5* encodes CLC-5 protein, a chloride proton exchanger expressed in proximal tubule cells and acid-transporting intercalated cells in distal nephron in the kidney [20]. The p.L503* mutation of *CLCN5* is close to a *CLCN5* p.G506E mutation, which belongs to class 1 mutations inducing defective protein processing [21]. The CLC-5 mutants are retained within the endoplasmic reticulum and targeted for degradation by quality control mechanisms [21]. Dent disease 2 is caused by pathogenic variants in the *OCRL* gene which maps on chromosome Xq25 and encodes a lipid phosphatase that hydrolyzes phosphatidyl-inositol 4,5-bisphosphate (PIP2) [5]. Previous research in zebrafish suggested that *OCRL1* was involved in the renal tubular endocytotic pathway: the accumulation of PIP2 resulting from decreased *OCRL1* activity was thought to alter endocytosis-related cell signaling, resulting in disruption of certain cell-cell contacts in the proximal tubule [22]. Impaired endocytosis might result in decreased proximal tubular reabsorption of parathyroid hormone (PTH). The high urine PTH would increase conversion of 25(OH)-vitamin D3 to 1,25 (OH)-vitamin D3, and further enhance intestinal calcium reabsorption and promote hypercalciuria (a major feature of Dent disease) [13, 16]. Nonsense mutations create premature termination codons (PTCs) in genes that in turn trigger nonsense-mediated mRNA decay to selectively and rapidly degrade PTC-bearing aberrant transcripts [23]. Decreased *OCRL1* activity resulting from the nonsense mutation in the *OCRL1* gene may disrupt the function of proximal tubule. A genetic analysis showed Case 2, who was initially diagnosed with Fanconi syndrome, carried the hemizygous deletion variant in the *OCRL1* gene and he was diagnosed as Dent disease 2 eventually. Therefore, genetic tests are necessary for a definite clinical diagnosis and for distinguishing Dent disease from Fanconi syndrome. Hamilton et al. first proposed that the *HNF4A* p.R85W (also described as p.R76W or p.R63W) mutation was a pathogenic variant specific for renal Fanconi syndrome [14]. Recent report indicated hyperinsulinemic hypoglycemia and maturity-onset diabetes of the young were due to *HNF4A* pathogenic variants [24, 25]. Case 3 was suspective of having Fanconi syndrome because of proteinuria, glycosuria, aminoaciduria and osteoporosis. The mutational analysis of this case revealed a p.R85W pathogenic variant in the *HNF4A* gene. Contrary to typical symptoms with Fanconi syndrome, Case 3 did not have metabolic acidosis or hyperchloremia. However, the child presented with glycosuria and hypoglycemia which was consistent with the consequence of *HNF4A* pathogenic variants [7, 25]. He fulfilled the criteria for Fanconi syndrome and might belong to an acquired type.

In conclusion, LMW proteinuria should be performed for patients with proteinuria to achieve early diagnosis and treatment. Since Dent disease may present with clinical features of Fanconi syndrome, Dent disease should be taken into consideration when the patients with Fanconi syndrome have LMW proteinuria and hypercalciuria. Definite diagnosis and identification requires further genetic analyses.

## Data Availability

All the data relevant to this report are included in the manuscript.

## Abbreviations

LMW proteinuria: Low molecular weight proteinuria
UTP: Urinary total protein
ACR: Albumin-to-creatinine ratio
α1-MG: α1-microglobin
UCa/Ucr: Urinary calcium to creatinine ratio
24-h Ccr: 24-hour creatinine clearance
eGFR: Estimated glomerular filtration rate
